# Histomorphometric comparison of the corpus cavernosum of rats submitted to euthanasia with ketamine and xylazine or isoflurane

**DOI:** 10.1590/ACB361103

**Published:** 2021-12-08

**Authors:** Isabella Mendes Procópio, Marco Aurélio Pereira-Sampaio, Waldemar Silva Costa, Francisco José Barcellos Sampaio, Diogo Benchimol de Souza

**Affiliations:** 1 Fellow Master degree. Urogenital Research Unit – Universidade do Estado do Rio de Janeiro - Rio de Janeiro (RJ), Brazil.; 2 PhD. Department of Morphology – Universidade Federal Fluminense - Rio de Janeiro (RJ), Brazil; 3 PhD. Urogenital Research Unit - Universidade do Estado do Rio de Janeiro - Rio de Janeiro (RJ), Brazil.; 4 PhD. Urogenital Research Unit - Universidade do Estado do Rio de Janeiro - Rio de Janeiro (RJ), Brazil.

**Keywords:** Euthanasia, Penis, Models, Animals, Rats

## Abstract

**Purpose:**

To compare the penile histoarchitecture of rats euthanized with isoflurane or with ketamine and xylazine.

**Methods:**

Fourteen male rats were divided into two groups: ISO, with animals euthanized with isoflurane; and K+X, with animals euthanized with ketamine (150 mg/kg) associated with xylazine (15 mg/kg). Immediately after the death, the penises were dissected, fixed in a 4% buffered formalin solution, and processed for histomorphometric analysis. The surface densities (Sv) of the corpus cavernosum structures (connective tissue, smooth muscle, sinusoidal space, and elastic fibers) were evaluated using Image J software. The distribution of collagen types I and III was qualitatively assessed. Statistical analyses were performed using the Student’s t test for data comparison, considering it statistically significant when p < 0.05.

**Results:**

Regarding the Sv of connective tissue, smooth muscle and sinusoidal space, there were no differences between animals in both groups. On the other hand, the animals euthanized with the association of ketamine and xylazine showed the Sv of elastic fibers 24.8% higher in relation to animals euthanized with isoflurane.

**Conclusions:**

The euthanasia method affected one of the morphological parameters of the rat penises. The choice of euthanasia method must be standardized to reduce bias and to obtain reliable and reproducible results.

## Introduction

Despite efforts to reduce the use of animals in experimentation, in many studies they are still necessary to obtain answers regarding biological and/or pathological processes^1^. Experiments often involve euthanasia of the animals, either for collection, to alleviate a condition of suffering, or to terminate the study. In this sense, anesthetic practices for performing euthanasia and its different methods are frequently studied, and recommendations for euthanasia change based on new information, always refining the experiments, and reducing the animals’ discomfort^2^.

The term euthanasia depicts the induction of death without pain or suffering. In general, euthanasia techniques must be effective in promoting death, producing rapid loss of consciousness, not causing terror or suffer to the animal, and being easy to apply^3^^,^ 4. However, one other utmost characteristic to maintain adequate experimental conditions is that euthanasia methods shall not produce parametric changes that may impair the study results and interpretations.

The methods are divided into two main ones: chemistry and physics^5^. When it comes to euthanasia by chemical methods, there is a risk of interference and confusion in the variables of interest, due to the use of different anesthetic and analgesic agents, with distinct anesthetic approaches, influencing comparisons of results and reproduction in different laboratories^6^^-^ 8.

When tissue collection and analyses are necessary, methods that reduce the occurrence of artifacts and ensure painless death induction should be prioritized^3^^,^ 9. Little information is available about tissue changes associated with euthanasia methods. Therefore, it is essential to know the differences between the types of anesthetics and euthanasia methods, seeking selection and standardization in the research work.

Several studies use the rat penis to study erectile dysfunction and other penile medical conditions. In most of these experiments, the organ is collected after euthanasia and analyzed histologically. However, no study investigated the hypothesis that different methods of euthanasia may be associated with some histomorphological variation of the corpus cavernosum.

Thus, the present study aimed to compare the histoarchitecture of the corpus cavernosum of rats euthanized with isoflurane or with the association of ketamine hydrochloride and xylazine hydrochloride (two of the most commonly used methods).

## Methods

This project was approved by the Animal Care and Use Committee of the Universidade do Estado do Rio de Janeiro (protocol 004-2019).

Fourteen male Wistar rats were used for this study. All animals were bred in our laboratory and kept in a room with controlled temperature (24°C ± 1°C) and an artificial light-dark cycle (lights on from 7 a.m. to 7 p.m.). Rats had free access to standard food and water. All experiments were performed according to national and international law for the scientific use of animals.

All animals were kept under standard conditions, without any intervention being performed up to the 16th week of life, when they were submitted to euthanasia. At that time, the animals were randomly divided into two groups of seven animals each. The K+X group was composed by rats euthanized by intramuscular injection of 10% ketamine hydrochloride (150 mg/kg, Cetamin, Syntec, Barueri, SP, Brazil) associated with 2% xylazine hydrochloride (15 mg/kg, Xilazin, Syntec, Barueri, SP, Brazil). The ISO group was euthanized by the administration of 2% isoflurane (Isoforine, Cristália, Itapira, SP, Brazil) vaporized in an induction chamber.

After euthanasia, the penises were collected, and the skin-denuded middle part of the penile shaft was fixed in 4%-buffered formaldehyde solution and processed for paraffin embedding. Penile cross-sections (5-μm thick) were obtained and used for histomorphometric evaluations.

Sections stained by Weigert’s Resorcin-fuchsin (with previous oxidation) were photographed under x600 magnification and used to analyze the surface densities (Sv) of the elastic system fibers. In other sections, stained by Masson’s trichrome and photographed under x400 magnification, the Sv of the sinusoidal space, connective tissue and smooth muscle of the corpus cavernosum were evaluated. In sections stained with picrosirius red and photographed under polarized light at x400 magnification, the distribution of collagen type I and type III was analyzed^10^. All photomicrographs were captured by a camera (DP70, Olympus, Tokyo, Japan) attached to the microscope (BX51, Olympus, Tokyo, Japan).

The Sv of the different structures were evaluated by the point-counting method^11^. Twenty-five photomicrographs of the corpus cavernosum were analyzed for each animal, for each structure. Briefly, a 100-point grid was superimposed over the images using the Image J software (National Institutes of Health, United States of America), and each point touching a structure of interest (elastic fibers, smooth muscle, connective tissue, or sinusoidal space) was counted to determine a percentual value which corresponded to the Sv of each structure.

Statistical analyses were performed using the GraphPad Prism 5 software, the Kolmogorov-Smirnov test to verify the normal distribution of data and the paired Student’s t test for data acquisition, considering p *<*0.05 as a significant result. All results are presented as mean ± standard deviation.

## Results

**Figure 1:**
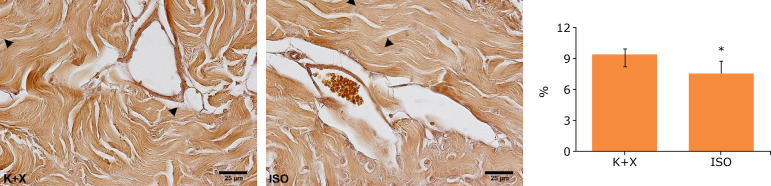
Photomicrographs of corpus cavernosum of rats submitted to euthanasia with ketamine and xylazine (K+X) or isoflurane (ISO); and graphic representation of surface density of elastic fibers results. Arrow heads indicates some elastic fibers. Weigert’s Resorcin-fuchsin (with previous oxidation), magnification x600.

There was significant difference between the groups regarding the Sv of elastic system fibers (p = 0.0057). Animals in the K+X group had 24.8% higher values in comparison to the animals in the ISO group (Fig. 1).

**Figure 2:**
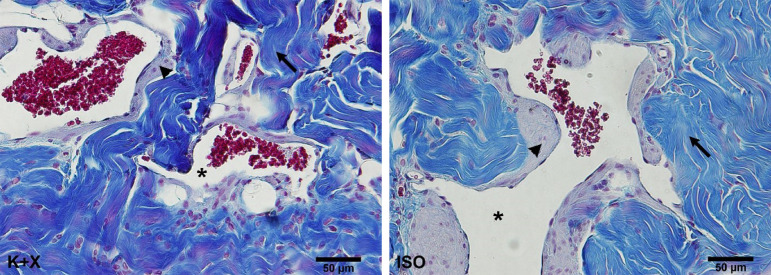
Photomicrographs of corpus cavernosum of rats submitted to euthanasia with ketamine and xylazine (K+X) or isoflurane (ISO). Arrows indicate the connective tissue; arrow heads point the smooth muscle fibers; asterisk indicates the sinusoidal space. Masson’s trichrome, magnification x400.

There were no significant differences in Sv of connective tissue, sinusoidal space, and smooth muscle between the groups (Fig. 2). All numerical data are shown in Table 1.

**Figure 3:**
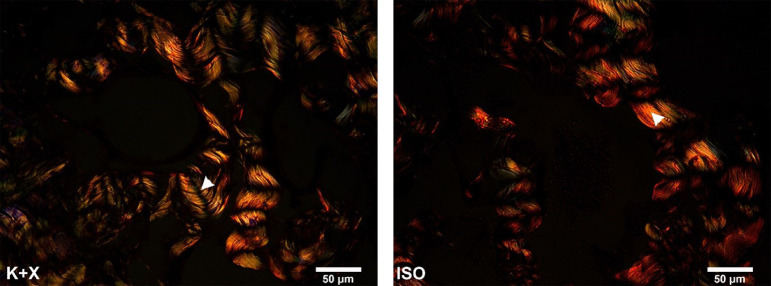
Photomicrographs of corpus cavernosum of rats stained with picrosirius red and observed under polarized light microscopy. Arrow heads indicate the collagen fibers. Magnification x400.

When comparing the different types of collagen, through a qualitative analysis, it was observed that both groups similarly showed predominance of type I collagen, without remarkable disparities among the groups (Fig. 3).

K+X: animals submitted to euthanasia with ketamine and xylazine; ISO: animals submitted to euthanasia with isoflurane.

**Table 1:** Histomorphometrical data of the corpus cavernosum of rats euthanized with ketamine and xylazine (K+X) or isoflurane (ISO)*.

	**K+X**	**ISO**	***p* value**
Sv of sinusoidal space (%)	18.63 ± 5.03	24.03 ± 4.47	0.1170
Sv of connective tissue (%)	72.08 ± 7.82	66.49 ± 5.40	0.2195
Sv of smooth muscle (%)	7.07 ± 1.38	8.42 ± 1.37	0.2747
Sv of elastic fibers (%)	9.42 ± 0.56#	7.53 ± 1.16	0.0057

Sv: Surface density;

* data expressed as mean ± standard deviation;

# statistically different from group ISO.

The data expressed in the table for each group was obtained through Kolmogorov-Smirnov test, to verify the normal distribution of data, and the paired Student’s t test for data acquisition, considering p < 0.05 as a significant result.

## Discussion

Little is known about the interference of euthanasia methods in the reproductive tract organs of laboratory animals. Histological and morphometric studies on the structure of the penis of rodents subjected to the effects of anesthetic agents were not found. Existing studies on the anesthetic and euthanasia protocol sought to investigate physiological and morphological parameters in other organs^3^^,^ 5 ^,^ 12 ^-^ 15. The present study is the first one to demonstrate and compare the effects of different euthanasia methods on the morphology of the rat penis.

The combination of ketamine and xylazine injection produces excellent anesthesia and analgesia, and its use is common as an anesthesia and euthanasia protocol. Ketamine is a non-competitive antagonist at the ionotropic glutamatergic receptor of N-methyl D-aspartate (known as NMDA). These are excitatory receptors involved in the enhancement of nociceptive processing, which has been shown to avoid sensitization to harmful stimuli during surgery. Ketamine is also associated to transient increases in systemic blood pressure, heart rate, cardiac output, sympathetic stimulation, and skeletal muscle tone^16^^,^ 17.

Xylazine is an α2 adrenergic agonist with analgesic, sedative, and centrally acting muscle relaxant properties. The action of xylazine in the activation of postsynaptic α2 receptors in peripheral vascular smooth muscle promotes an initial increase in blood pressure causing peripheral vasoconstriction^18^, promoting smooth muscle contraction in the corpus cavernosum^19^. The administration of xylazine is considered safe when used alone or in combination with other anesthetic and analgesic agents, such as ketamine, in animal research^8^.

Isoflurane is a halogenated methylethyl ether that has anesthetic properties defined by amnesia, immobility, analgesia, and unconsciousness, producing moderate depression of the respiratory and cardiovascular system, with increased heart rate^20^. It is also associated with vascular smooth muscle relaxation.

According to Baneux *et al*.^21^, rabbits that were anesthetized with a combination of ketamine and xylazine and subsequently euthanized with ketamine did not demonstrate histopathological changes in the tissues that were evaluated in the study (brain, heart, lungs, liver, spleen, kidneys, stomach, small and large intestines, urinary bladder, ovary, uterus, and cervix). By the other hand, Koehn *et al*.^22^ showed that anesthesia with a combination of ketamine and xylazine caused histological alterations in the cornea of mice.

The proper choice of anesthetics in experiments is challenging and must be carefully adapted depending on the model and the end points, avoiding directly affecting the tissue’s viability or parameters^2^.

Modifications in the proportion of one of the components of the corpus cavernosum (elastic fibers) were identified in this study. However, the general histoarchitecture of the corpus cavernosum did not change when using isoflurane or the association of ketamine and xylazine as agents for euthanasia.

In the present study, the different euthanasia protocols presented similar outcomes for the smooth muscle area density, sinusoidal space, and connective tissue of the corpus cavernosum of rats. Thus, if these are the only parameters to be investigated, there would be no difference that would justify or condemn the use of one of the methods.

On the other hand, if elastic fibers are the object of study, attention should be paid to standardize the method of euthanasia. According to the analyses of this study, rats killed with ketamine and xylazine, compared to animals killed with isoflurane, had higher density of elastic fibers, while the other components of the corpus cavernosum have not been shown to undergo changes. The reason for this is still unknown. The role of elastic fibers in the erection mechanism is not clear, and there is controversial information about the relationship between elastic fibers and erection^23^. Changes in the number of elastic fibers have already been demonstrated in some pathological situations (erectile dysfunction and diabetes mellitus)^24^^,^ 25, however it has never been associated with euthanasia protocol.

It is supposed that, to have a production or depletion of connective tissue, elastic fibers or smooth muscle fibers in penile tissue, there should be more time (weeks or months) from the studied stimuli. For such a short-term influence of the studied treatment (as observed in the present study) on the elastic fibers, probably there was no turnover of this component. One hypothesis to explain this observed difference is that the used anesthetic agent could promote different muscle relaxation and/or fluid distribution among the tissues. The higher smooth muscle relaxation promoted by isoflurane could lead to augmented smooth muscle Sv and sinusoidal space Sv (by non-significant levels), reducing the proportional connective tissue Sv (also by a non-significant level). As elastic fibers are part of the connective tissue, this component proportional area was also reduced (what was statistically confirmed).

The present study has some limitations that must be considered. Different methods of euthanasia (rather than the studied ones here) are used, and the information of the present study is limited to only two methods. Further, other parameters (both morphological and physiological) that were not analyzed could be affected by the euthanasia method used.

Future studies should be carried out to add knowledge about possible changes in the corpus cavernosum under the effect of anesthetic agents used for euthanasia, thus offering more information on the standardization of the model to be used and the protocol to be adopted.

## Conclusion

The agent used to perform euthanasia in rats interferes on the Sv of elastic fibers. Although the other parameters have not been changed, the importance of standardizing the euthanasia method in experiments whose histology of the corpus cavernosum will be analyzed should be highlighted.
